# Cancer of the endometrium: current aspects of diagnostics and treatment

**DOI:** 10.1186/1477-7819-2-24

**Published:** 2004-07-21

**Authors:** Karsten Münstedt, Phillip Grant, Joachim Woenckhaus, Gabriele Roth, Hans-Rudolf Tinneberg

**Affiliations:** 1 Department of Obstetrics and Gynecology, Justus-Liebig-University Giessen, Klinikstrasse 32, D 35385 Giessen, Germany; 2 Department of Psychology, Justus-Liebig-University Giessen, Otto-Behagel-Str. 10F, D 35394 Giessen, Germany; 3 Institute of Pathology, Justus-Liebig-University Giessen, Langhansstrasse 10, D 35385 Giessen, Germany

## Abstract

**Background:**

Endometrial cancer represents a tumor entity with a great variation in its incidence throughout the world (range 1 to 25). This suggests enormous possibilities of cancer prevention due to the fact that the incidence is very much endocrine-related, chiefly with obesity, and thus most frequent in the developed world. As far as treatment is concerned, it is generally accepted that surgery represents the first choice of treatment. However, several recommendations seem reasonable especially with lymphadenectomy, even though they are not based on evidence. All high-risk cases are generally recommended for radiotherapy.

**Methods:**

A literature search of the Medline was carried out for all articles on endometrial carcinoma related to diagnosis and treatment. The articles were systematically reviewed and were categorized into incidence, etiology, precancerosis, early diagnosis, classification, staging, prevention, and treatment. The article is organized into several similar subheadings.

**Conclusions:**

In spite of the overall good prognosis during the early stages of the disease, the survival is poor in advanced stages or recurrences. Diagnostic measures are very well able to detect asymptomatic recurrences. These only seem justified if patients' chances are likely to improve, otherwise such measures increases costs as well as decrease the patients' quality of life. To date neither current nor improved concepts of endocrine treatment or chemotherapy have been able to substantially increase patients' chances of survival. Therefore, newer concepts into the use of antibodies e.g. trastuzumab in HER2-overexpressing tumors and the newer endocrine compounds will need to be investigated. Furthermore, it would seem highly desirable if future studies were to identify valid criteria for an individualized management, thereby maximizing the benefits and minimizing the risks.

## Incidence and mortality

**Figure 1:**
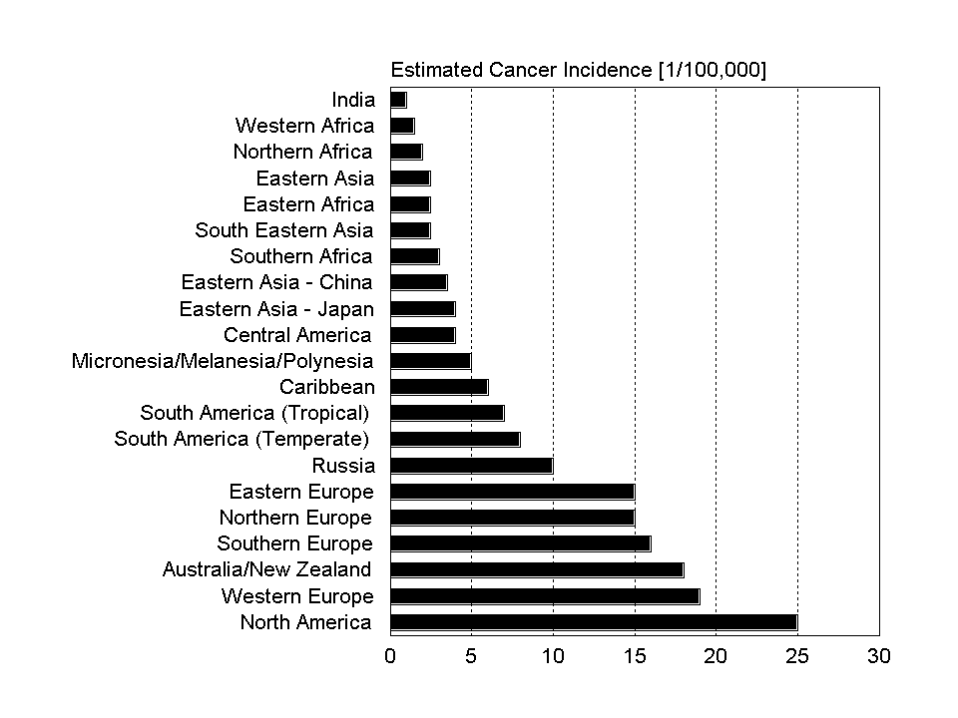
Estimated endometrial cancer incidences throughout different regions of the world [1].

**Figure 2:**
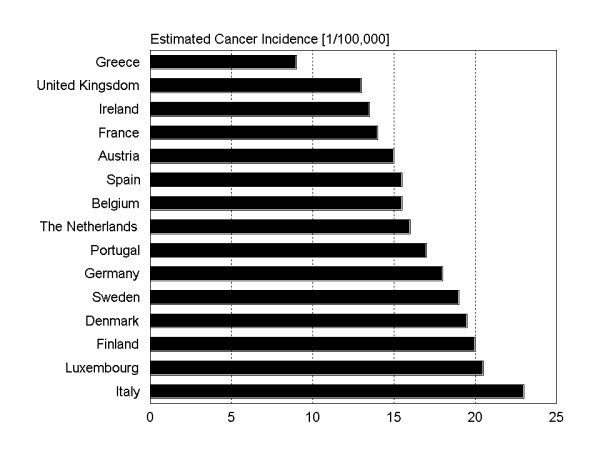
Estimated endometrial cancer incidences throughout different countries in Europe [1].

Nearly 170,000 new cases of endometrial carcinomas were estimated worldwide in 1997 [1]. However, incidences throughout different regions of the world vary considerably. Compared to Africa and Asia having the lowest rates of incidence, Western Europe, USA and Canada are shown to have the highest incidence worldwide (Figure 1). As shown in figure 2, even within Europe the incidence rates are very heterogeneous. In some of these countries, e.g. Germany, endometrial carcinoma is the most common among genital carcinoma [2].

**Figure 3:**
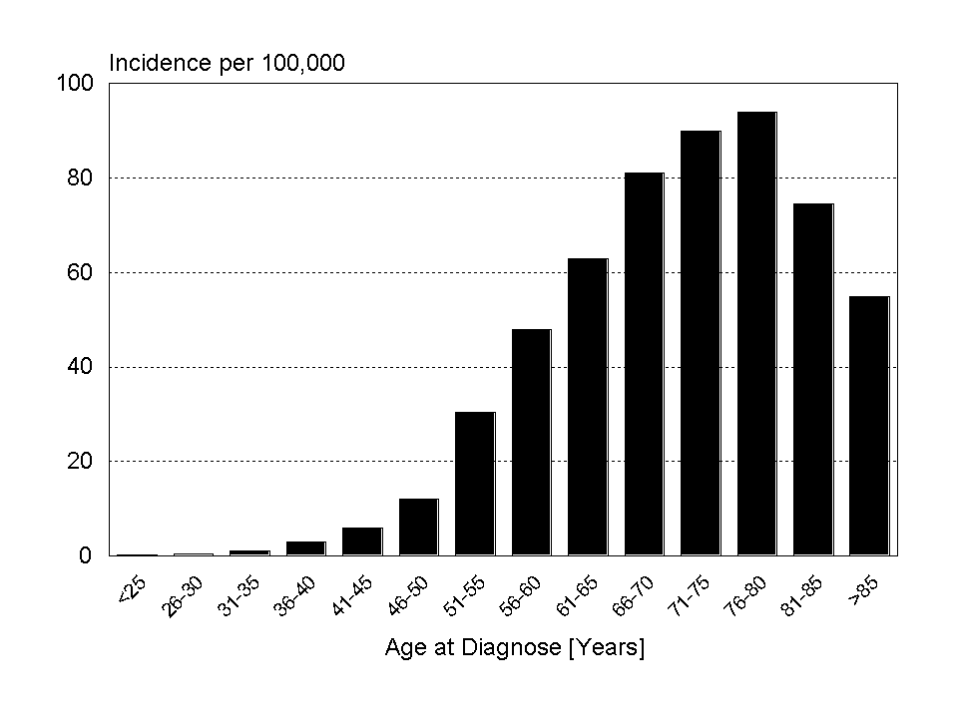
Age dependent incidence of endometrial cancer in Germany. Robert-Koch-Institute at:

Endometrial carcinomas occur in advanced age (postmenopausal). The age-related incidence for Germany is shown in Figure 3. The overall increase in the incidence of this disease during the last decades is mainly related to higher life expectancy within the developed world.

## Etiology and risk factors

The etiology of the endometrial carcinoma is not fully understood. Most cases appear sporadically whereas about 10% are hereditary. Chief among the latter is the autosomal dominantly inherited hereditary non-polyposis colorectal cancer (HNPCC) [3-5]. The risk of developing endometrial cancer is believed to be ten times higher for women carrying the gene compared to the general population [6]. The likelihood of a synchronous or metachronous development of endometrial carcinomas is, however, higher for patients with breast, ovarian, and non-hereditary colorectal cancer [7].

Within the current concept of multi-step progression of normal cells to malignancy, recent molecular work has identified several gene alterations important for tumor development. In summary, mutations and amplifications of oncogenes K-ras and HER2/neu, mutations or deletions of tumor suppressor genes p53, p21, p16, and pTEN/MMAC1 as well as impaired DNA repair functions through mutations of hMLH1, hMSH2, and hMSH6 have been connected with the development of endometrial carcinomas [8].

Environmental, dietary and hormonal factors as well as an aging female population have been attributed to an observed increase of endometrial carcinomas over the past few years. Epidemiologic studies have observed correlations between the incidence of endometrial cancer and the usage of estrogens, especially when applied to alleviate perimenopausal and postmenopausal symptoms. Therefore, it appears that estrogen plays a key role in the development and progression of endometrial carcinomas.

There is also convincing evidence that high body mass increases the risk of endometrial carcinomas. Current estimations figure that about 40% of these relate to excess body weight [9]. A plausible biological explanation for obesity influencing the risk of endometrial cancer is found in increased aromatization of androstendione to estrone in adipose tissue [10]. Hyperadrenocorticism, which is more common in obese individuals, also disturbs the estrogen metabolism. The correlation between obesity and hyperadrenocorticism is possibly increased by hyperinsulinism. This also explains the higher frequency of endometrial carcinomas in combination with diabetes mellitus. A high intake of saturated fat may also increase the risk, whereas high consumption of vegetables and fruits may do the opposite [1,11]. Due to the fact that most factors relate to prolonged or intensive exposure to estrogen (hyperestrogenism), this may likely be identified as the underlying concept leading to the development of endometrial cancer. In this process estrogens are believed not to act as carcinogenic agents, but as promoters of carcinogenesis. A state of hyperestrogenism may be caused by exogenous or endogenous factors and may relate to reproductive factors, estrogen exposure or menopausal years [9,12,13]. Several endogenous risk factors are also associated with the increase of endometrial cancer risk e.g. early menarche, late menopause (2.4-fold), nulliparity (2-fold), the polycystic ovarian syndrome, diabetes mellitus (2.8-fold), high blood pressure (1.5-fold), obesity (up to 10-fold), other tumors with estrogen production, atypical endometrial hyperplasia (see Table 1) and the aforementioned inherited forms of colorectal cancer (10-fold). Basically two hypotheses endeavor to explain the protective effect of high parity: The first one assuming the mechanical removal of premalignant and malignant cells with each delivery, the second describing a protective effect of high progesterone values during pregnancy. It is most likely for both hypotheses to be correct to some extent, whereas a large population based study from Finland favors the second hypothesis [14]. An additional factor may be anovulatory ovarian insufficiency, especially in patients with polycystic ovaries (PCO syndrome). As these patients have a persistent progesterone deficiency due to non-formation of a corpus luteum, they lack this important protective mechanism. Endocrine risk factors also include hormone producing ovarian tumors. This goes for estrogen as well as androgen producing tumors since androgens may be converted to estrone in adipose tissue.

**Table 1:** Type of endometrial hyperplasia and rate of progression to cancer [20].

Type of hyperplasia	Rate of progression
Simple (cystic without atypia)	1
Complex (adenomatous without atypia)	3
Atypical	
Simple (cystic with atypia)	8
Complex (adenomatous with atypia)	29

Previous irradiation of the pelvis, estrogen replacement therapy (HRT) (especially unopposed HRT) and tamoxifen therapy represent exogenous risk factors. Prolonged and exclusive intake of synthetic estrogens is associated with an up to 15-fold higher incidence of endometrial carcinomas [15]. Similarly, some selective estrogen receptor modulators (SERMs) and especially tamoxifen may have an estrogenic, proliferative effect on the endometrium. Thus tamoxifen therapy is frequently associated with polyps, hyperplasia and carcinomas of the endometrium [16,17]. Tamoxifen seems to induce a 6.4-fold increase in the risk for endometrial cancer [18]. On the other hand, cyclic application of estrogens combined with progestins does not increase endometrial cancer incidence, e.g. hormonal contraception (combined preparations) and may even reduces the risk of endometrial carcinomas by up to 50% (see prevention). In essence, hyperestrogenism will lead to stronger proliferation, thereby causing hyperplasia which may gradually acquire more and more cellular atypia (atypical hyperplasia) and later transform into an endometrioid adenocarcinoma.

Recently a case-control study identified antipsychotic drugs as being a great risk factor for endometrial cancer [19]. These findings may also be explained to some extent by the side effects of the drugs (obesity, insulin resistance, amenorrhea, low gonadal steroids leading to hyperprolactinemia).

## Precancerosis

In some cases endometrial cancer develops from atypical endometrial hyperplasia. The likelihood of this happening correlates with the degree of hyperplasia (Table 1) [20]. According to the World Health Organization (WHO) classification, endometrioid carcinomas are divided into simple and complex forms, each with and without atypia [21]. This classification has also been accepted by the International Society of Gynecological Pathologists.

Endometrial hyperplasia is regarded as a preliminary stage of endometrioid carcinomas. Serous and clear cell carcinomas are on the other hand frequently associated with an atrophic endometrium [22]. A precursor of the serous carcinoma and possibly of some clear cell carcinomas is the endometrial intraepithelial carcinoma (EIC) [22,23].

## Prevention

To prevent an outbreak of the disease in menopausal and postmenopausal women long-term estrogen replacement therapy (treatment of menopausal symptoms, osteoporosis etc.) should be supplemented by intermittent application of gestagens. A recent study suggested the use of intrauterine devices (IUD) also possibly reducing the risk for endometrial cancer due to improved elimination or decrease of hyperplastic endometrial cells [24]. Numerous studies have shown that cigarette smoke reduces the risk of an endometrial carcinoma for women after the menopause, although it may increase the risk for premenopausal women [25]. The greatest reduction of risk was found in obese, multiparous women who did not receive hormone treatment [26]. Women in advanced stages of disease (stages II – IV), however, were found more likely to smoke than women in early stages (0 – I). This may reflect a smoking-related decrease in the incidence of early-stage tumors as well as an increase in tumor invasiveness and metastases [27]. The risk of endometrial carcinomas may be reduced significantly by prolonged progestin therapy every month (for 10 days) alone or in combination with estrogen [28]. Since progestins are known to act as cofactors of cancerization in breast and cervical cancer such concepts are better interpreted cautiously [29]. Therefore hormonal preventive concepts need to undergo a general assessment of benefits and risks. To sum up, apart from excess body fat reduction and omission of unnecessary estrogen therapy, there appears to be no reasonable way of preventing endometrial carcinomas.

## Early detection

To date there is no procedure that seems appropriate as a screening method for early detection of endometrial carcinomas. Current guidelines of the American Cancer Society suggest informing patients of risks and symptoms involved with endometrial cancer and furthermore firmly emphasize the importance of reporting unexpected bleeding or spots to their physician [30]. The fact of most endometrial carcinomas (with the exception of the rare serous and clear cell types) showing these kinds of preliminary symptoms leads to the diagnosis of over 75% of cases while still in stage I [7]. A recent case-report on the usage of the Mirena^® ^intrauterine system maintains that irregular menstrual bleeding should not be treated simply with this system without prior diagnostic [31].

The necessity of finding a screening method is discussed controversially in this context. So far, most epidemiological studies have failed to show significant effects of screening on mortality.

In some cases the Pap smear may lead to the diagnosis of endometrial cancer. However, in many cases cells from inside the uterus are not assessed by the sampling procedure. Positive cervical cytology was found to correlate with nodal spread in 91% of cases, whereas the risk of lymph node spread in patients with normal cervical cytology was estimated at around 2% [32]. It would seem too early to suggest that this could help to reach a decision on the necessity of lymphadenectomy. Maybe the ThinPrep Pap tests will be able to allow further conclusions in the future [33].

Transvaginal ultrasound has also been suggested as a potential means of early detection of endometrial carcinomas. A recent meta-analysis involving 9,031 patients and covering 57 separate studies on the diagnostic accuracy and positive predictive power of endometrial thickness measurement by pelvic ultrasound in patients with postmenopausal bleeding concluded that these measurements cannot solely be used to accurately rule out endometrial pathology. Measurement of both endometrial layers of ≤ 5 mm coincides with endometrial pathology in only 2.5% [34]. Other studies have used saline infusion sonography and color Doppler sonography to differentiate between endometrial cancer, endometrial hyperplasia, fibroids, endometriosis, myoma or tamoxifen induced endometrial thickness [35-42]. A recent comparison of saline infusion sonography and office hysteroscopy revealed similar ratings of patients' pelvic pain during the procedures. Sensitivity and specificity coefficients as well as negative and positive predictive values were higher for the office hysteroscopy [43].

It seems that biopsy remains the only accurate way of diagnosing endometrial cancer [44]. Under optimal circumstances the gynecologist will remove a tissue sample from the uterine lining under hysteroscopic control [44-47]. If hysteroscopic control is neglected, the false negative rates for dilatation and curettage (D&C) will range between two and six percent, thereby emphasizing the limitations of D&C alone [45,48-50].

Newer methods, like magnetic resonance imaging (MRI), positron emission tomography (PET), intraoperative ultrasound or three dimensional sonography are not likely to gain importance with respect to the diagnosis or early diagnosis of the disease [51-56]. It is, however, reasonable to believe that they are likely to deliver more information about the invasion depth of the myometrium or lymphatic metastases [52,55,57].

## Classification of endometrial carcinoma

The term endometrial carcinoma describes a variety of different tumor types originating from the inner ling of the uterus. Many authors differentiate between two basic types, which may be divided into estrogen-dependent and estrogen-independent types or tumors with favorable or unfavorable prognosis [58]. Although there are no cross-sectional studies comparing tumors from various ethnic groups and significant differences in tumor biology the frequency of tumors of a certain category in various geographical areas have been assumed. This may explain why mortality in the USA is higher in black women than in white women (5.8 deaths per 100,000 persons in black women vs. 3.1 deaths per 100,000 persons in white women) [59]. The different categories may be summarized as follows:

1. It comprises of estrogen related tumors occurring in younger, perimenopausal women. These tumors are often said to be highly differentiated, mainly adenocarcinomas with positive steroid hormone receptor status (ER, PR), the known risk factors (estrogen etc.) originating from atypical endometrial hyperplasia. The patients in this group have a longer history, lower grade tumors, less myometrial invasion and low potential for lymphatic spread. They may be associated with concomitant carcinomas of the ovary, breast and colon and respond to progestin therapy. The overall prognosis is generally favorable.

2. This category is comprised of tumors of patients with shorter history, higher grade tumors, deeper myometrial invasion and high risk of lymphatic spread. The tumors will not respond to progestin therapy and there are no associated tumors. Histologically these tumors are identified as serous carcinomas or clear cell adenocarcinomas. The prognosis of patients with this type of tumor is poor.

3. Findings of carcinomas in atrophic endometrium being associated with an intermediate prognosis lead to the suggestion of a third category. In these cases the endometrioid carcinomas are not likely to be estrogen-related [21].

4. It was furthermore suggested to incorporate endometrial neoplasia originating from an inherited predisposition into a fourth category. These types of tumors tend to develop about 15 years earlier and are associated with a favorable prognosis. Most patients in this category have a hereditary non-polyposis colorectal carcinoma syndrome (HNPCC).

## Histological types according to the WHO classification

The following section will briefly describe the most important characteristics of endometrial carcinomas. Detailed descriptions may be found in specialized pathological textbooks e.g. Anderson et al. [21].

• *Endometrioid adenocarcinoma*: The endometrioid adenocarcinoma, whose glands resemble those of the normal endometrium, is the most common type (60 – 80%). This type is considered part of the 1^st ^category

• *Endometrioid adenocarcinoma with squamous cell differentiation *(adenoacanthoma, adenosquamous carcinoma): Approximately 25% of endometrioid adenocarcinomas display partial squamous differentiation. The squamous elements are interpreted as *terminally differentiated *indicating that the tumor is incapable of independent growth. Prognosis depends on the glandular components of the lesion. Highly differentiated tumors (adenoacanthomata) have a favorable prognosis, whereas poorly differentiated tumors (adenosquamous carcinomas) have an unfavorable course.

• *Serous adenocarcinoma*: This tumor type is similar to the serous ovarian carcinoma. It is characterized by aggressive growth and poor prognosis. Lymphogenic and hematogenic metastases are usually already present at the time of diagnosis. Nearly all tumors are poorly differentiated. Serous adenocarcinomas belong to the 2^nd ^category of endometrial cancer.

• *Clear cell adenocarcinoma*: This type comprises 3 to 6% of all endometrial carcinomas. Like the serous adenocarcinoma, clear cell adenocarcinomas tend to progress rapidly. They share the 2^nd ^category.

• *Mucinous adenocarcinoma*: Diagnosis is based on the presence of mucus within the tumor cells. Purely mucinous carcinomas are rare, although a mucinous component within endometrioid carcinomas is more common. The tumors are usually highly differentiated and have a good prognosis. It is important to exclude a primary mucinous adenocarcinoma originating from the endocervix, spreading into the uterine body. Mucinous adenocarcinomas are part of the 1^st ^category.

• *Squamous cell carcinoma *(2^nd ^category): A very rare entity associated with very poor survival. Primary squamous cell carcinomas of the uterine cervix should be ruled out. Adjuvant platinum-based radiochemotherapy may result in improved survival.

• *Mixed carcinoma*: A carcinoma composed of two or more different non-squamocellular components with each component occupying at least 10% of the tumor. Prognosis varies, e.g. in case of a serous component, the prognosis is poor.

• *Undifferentiated carcinoma*: A rare carcinoma without glandular, squamous or sarcomatous differentiation. Prognosis is unfavorable.

• Rare types of endometrial carcinomas: small cell carcinomas, microglandular adenocarcinomas, signet-ring cell carcinomas, transitional cell carcinomas, glassy cell carcinomas, mucinous adenocarcinomas of the intestinal type, lymphoepithelioma-like carcinomas, and endometrial adenocarcinomas with trophoblastic differentiation have been reported.

## Grading

The tumor grading is a highly significant prognostic parameter although it is subjective with a considerable inter-observer and intra-observer variability. It is determined by the percentage of non-squamocellular, solid portions as follows: G1: 5% and less, G2: 5–50%, G3: more than 50%. A significant nuclear atypia increases the grade of differentiation by one grade.

## Preoperative diagnostic procedures

After histological confirmation of an endometrial carcinoma clinical palpation and vaginal sonography should be performed. Several additional examinations have been suggested: Rectoscopy, cystoscopy, computed tomography and/or MRI. They may be omitted in clearly diagnosed early cases but are strongly recommended in advanced stages. Recent studies indicate some value of tumor marker diagnostics. At a cut-off level of 40 U/ml elevated CA125 serum levels indicate nodal metastases with a sensitivity of 77.8% and a specificity of 81.1% [60,61].

## Surgical therapy

**Figure 4:**
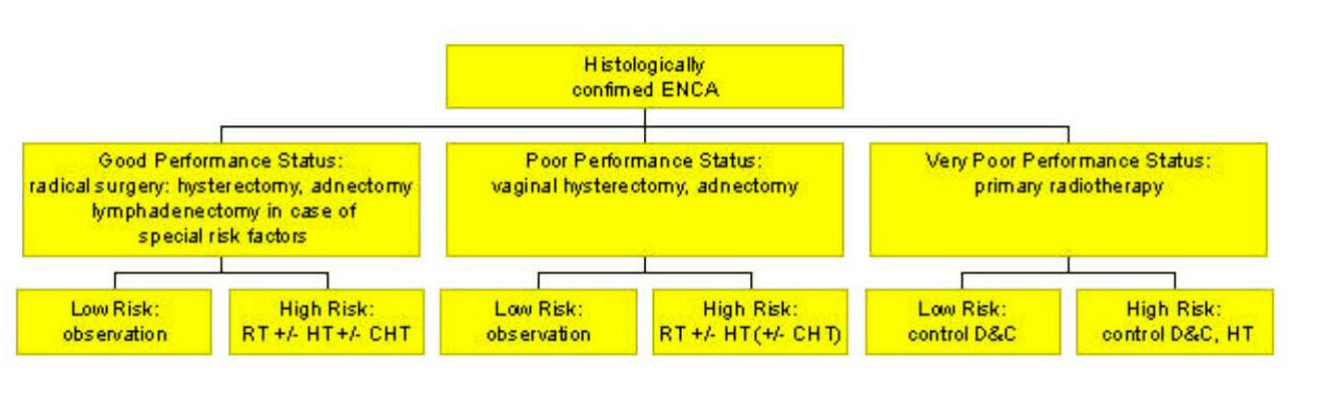
Decision tree concerning the primary treatment of endometrial carcinoma.Legend: RT = radiotherapy; HT = endocrine treatment; CHT = chemotherapy; D&C = dilatation and curettage

In all stages of endometrial carcinomas, surgery is the primary treatment of choice. Preoperative intracavitary radiation treatment, often recommended in earlier times, is not considered advisable any more since the information about the depth of myometrial invasion and thus information on an important prognostic factors is lost [62]. A study indicates that the Maylard-type incision is superior to transverse (Pfannenstiel-type) or longitudinal incision [63]. If possible, an abdominal hysterectomy with removal of the adnexa and a peritoneal lavage should be performed. After removal of the uterus, the depth of tumor invasion into the myometrium has to be determined to estimate the probability of extrauterine spread. If the myometrium is infiltrated to more than 50%, a pelvic and paraaortic lymphadenectomy should be performed. For appropriate staging, more than 20 lymph nodes should be dissected [64]. But also in cases of additional adverse prognostic factors (poor grading, lymphangiosis, see below), pelvic and paraaortic lymphadenectomy are recommended by many [65]. A decision tree on primary therapy is given in figure 4. Unfortunately, studies found no correlation between depth of invasion, histological grade, cervical invasion, peritoneal cytology, menopausal status, preoperative serum CA125 level or primary tumor diameter. Only lymphvascular space involvement (P < 0.0001) was significantly correlated to pelvic lymph node metastases, which lead the authors to the conclusion that all patients should undergo extended surgical staging, except when clinical or operative factors increase patients' morbidity [66]. Lymphadenectomy may be omitted in cases of more favorable prognosis.

There is some controversy on the value of radical hysterectomy in stage II carcinomas. While several earlier studies advocated this procedure [67-70] a recent study reported no prognostic advantage [71]. Maybe only the patients with stage IIb tumors and clinically evident tumor infiltration profit from radical hysterectomy. This requires further investigation.

Complications after pelvic lymphadenectomy may be reduced by omentoplasty and omentopexy [72]. In advanced stages of the disease complete removal of all tumor sites is warranted. In case of serous histology and peritoneal spread, some authors also advocate omentectomy. A recent study indicates that optimal cytoreduction results in a significant survival benefit for stage IVB endometrial cancer patients with a reasonable surgical morbidity rate [73].

Vaginal hysterectomy as primary treatment of endometrial cancer has also been investigated especially in medically compromised women [72,73]. Such approaches may be combined successfully with laparoscopically assisted radical vaginal hysterectomy [74-77].

The aforementioned basic goals of surgery (hysterectomy, removal of the adnexa and lymphadenectomy in stages Ic and higher) should be reached especially in medically fit patients, because treatment along the recognized guidelines has been found to be prognostically favorable. Some studies, however, have reported the standards mentioned in many cases as not having been realized [78-80]. As shown in Italy there seems to be certain reluctance towards bringing current topics discussed in literature into practice [81].

The problem mentioned may partly be due to the fact that the current guidelines are insufficiently supported by randomized surgical trials. Interestingly the COSA-NZ-UK Endometrial Cancer Study Group trial showed that lymphadenectomy showed no advantage for endometrial cancer if primary surgery was followed by adjuvant radiotherapy [82]. Therefore studies on all surgical aspects are warranted. This also includes newer surgical approaches which await further evaluation in prospective studies.

## Staging

Endometrial carcinomas are staged surgically. Procedures previously used for determination of stages, such as fractional dilatation and curettage to differentiate between stage I and II, are no longer applicable unless the patient is to be treated by primary radiation therapy. The old (1971) and new (1988) staging system of the International Federation of Gynecology and Obstetrics (FIGO) are shown in table 2. The prognostic importance of adequate surgical staging was recently demonstrated [83].

**Table 2:** Tumor classification of the international Federation of Obstetrics and Gynecology (FIGO). The surgical staging system is obligatory unless patients are to undergo primary radiotherapy when the older clinical staging system may be used.

Stage	Stage – Clinical Staging		Stage – Surgical Staging	
**I**	I	Carcinoma confined to corpus	Ia	Tumor limited to endometrium
	Ia	Length of uterine cavity ≤ 8 cm	Ib	Invasion ≤ 1/2 myometrium
	Ib	Length of uterine cavity > 8 cm	Ic	Invasion > 1/2 myometrium
**II**	II	Carcinoma involves corpus and cervix	IIa	Endocervical glandular involvement only
			IIb	Cervical stromal invasion
**III**	III	Carcinoma extends outside uterus but not outside the true pelvis	IIIa	Tumor invades serosa or adnexa or positive peritoneal cytology
			IIIb	Vaginal metastasis
			IIIc	Metastases to pelvic or para-aortic lymph nodes
**VI**	IV	Carcinoma extents outside true pelvis or involves bladder or rectum	IVa	Tumor invades bladder, bowel mucosa, or both
			IVb	Distant metastases, including intra-abdominal and/or inguinal lymph nodes

## Prognostic and predictive factors

Tumor stage, patient age, histologic type and grade, hormone receptors and DNA ploidy represent the traditional prognostic factors. Respective of the response to progestin therapy steroid hormone receptors may also be regarded a predictive factors in recurrent and advanced disease [8]. The strong prognostic impact of tumor stage is underlined by the cumulative 5-year survival rates (surgical/pathological staging) which are 85% for stage I, 70% for stage II, 49% for stage III and 18% for stage IV disease. Obviously both major factors which make up the staging system of endometrial carcinomas, depth of invasion into the myometrium and lymph node status, are major prognostic factors [84,85]. A recently published analysis draws the attention to lymphvascular space involvement and suggests that its presence should indicate lymphadenectomy or adjuvant therapy [86]. Lymphvascular space involvement was also closely linked with advanced stage (unpublished observation). Furthermore, several additional prognostic factors have been suggested: Nulliparity, high tumor cell proliferation (KI-67), high tumor vessel density (angiogenesis), oncogene amplification or overexpression (HER2/neu, K-ras) and alterations of tumor suppressor genes (PTEN, p53, p21, p16) are believed to be associated with adverse prognoses. Especially Ki-67 could be of greater importance seeing that this parameter proved to be independent in multifactorial analyses in a prospective study [87].

There is a divergence of opinion concerning the value of a positive peritoneal cytology as an independent prognostic factor [88]. In stage I, depth of myometrial invasion, vascular invasion, mitosis count and progesterone receptor negativity are statistically significant prognostic factors [89,90]. Overexpression of p53 is observed in approximately 20% of all endometrial carcinomas and in up to 90% of serous carcinomas [91]. The plasminogen activator inhibitor type 2 has been discussed as a possible independent prognostic marker [92]. Overexpression of the oncogene Her-2/neu is significantly more common in advanced than in early stages. Finally, ploidy status is possibly an independent prognostic factor [93], with aneuploidy being mainly associated to prognostically unfavorable serous, clear cell and poorly differentiated carcinomas. There are, unfortunately, still too many controversies to draw final conclusions or even to make suggestions on factors to be determined routinely.

## Radiotherapy

### Primary radiotherapy

In medically compromised women, primary irradiation may be suitable. Analyses from Germany show that approximately 20% of all patients undergo primary radiotherapy [7,64]. There are three possible approaches: The after loading technique alone, a combination of after loading and percutaneous techniques or by percutaneous radiotherapy alone. The literature also reports on considerable variation in the number of fractions and the doses of each fraction [64]. In case of brachytherapy only 5 × 8 Gy or 8 × 5 Gy may be applied. Combined therapy usually delivers 50 Gy percutaneously with partial blocking of the bladder and intestines after 24 and 30 Gy and 2 to 5 fractions via brachytherapy. Brachytherapy may also be applied before commencement of percutaneous therapy. The selected dose may be applied by a variety of techniques e.g. by Heyman capsules, double-rod-shaped applicators, indwelling applicators etc. [64]. An appropriate applicator should ensure adequate irradiation of the entire uterus [94]. Nowadays, computer controlled treatment planning allows optimal treatment planning and an individual adaptation of the dose distribution to the uterine cavity. This may vary between patients of course.

**Figure 5:**
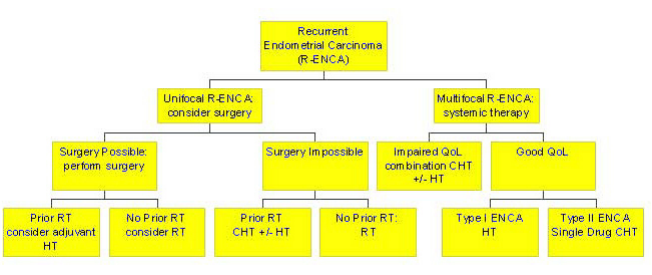
Decision tree concerning the treatment of recurrent endometrial carcinoma.Legend: ENCA = endometrial carcinoma; QoL = Quality of Life; RT = radiotherapy; HT = endocrine treatment; CHT = chemotherapy;

Only few clinical trials on primary radiotherapy have been performed. The results suggest that dividing doses into smaller fractions allows better tumor control and has less side effects [95]. The old question on high-dose-rate or low-dose-rate after loading still remains unsolved. There is still limited data on the efficiency of primary radiotherapy in endometrial carcinomas. An analysis of 154 patients having undergone primary radiotherapy at our department showed that local recurrences are more common in this group compared to surgery and adjuvant radiotherapy. The rates of recurrences are stage dependent: 23.3% vs. 13.2% in stage I and 39.2% vs. 25.9% in stage II [7]. As shown in figure 5 there is a significant difference in overall survival between both groups. Thus, primary radiotherapy represents an effective but suboptimal measure for this group of patients being in generally poorer physical condition.

### Adjuvant postoperative irradiation

Most studies indicate that advance age, grade 3 histology or deep myometrial invasion relate to a higher risk of disease recurrence. Therefore adjuvant radiotherapy seems important in this subset. External beam radiation should also be considered in cases of multiple infiltrated lymph nodes (> 5). But many questions remain unanswered and the value of postoperative irradiation is still under debate. Survival rates of patients with early stage disease are excellent, no matter whether they underwent extended-surgical staging with more restricted use of the adjuvant therapy or simple hysterectomy bilateral salpingoophorectomy with more frequent use of adjuvant radiotherapy [96]. Prospective-randomized trials have so far only demonstrated improved local control yet no overall survival benefit, and have higher rates of treatment related complications [97]. This accords to larger retrospective analyses, most likely due to the fact that the majority of these recurrences can be salvaged through radiation therapy [98]. With respect to the importance of the problem, trials to evaluate the therapeutic benefit of adjuvant radiotherapy in the several subsets of patients at higher risk are warranted.

The American Brachytherapy Society has now issued recommendations for brachytherapy for carcinomas of the endometrium [94]. According to these, the applicator selection should be based on patient and target volume geometry, the dose prescription point should be clearly specified and the treatment plan should be optimized. For intravaginal brachytherapy selection of the largest diameter applicator is to ensure close mucosal apposition. Finally, doses should be reported both at the vaginal surface and at 0.5-cm depth irrespective of the dose prescription point.

## Adjuvant medical treatment

The data on adjuvant medical treatment is not conclusive. Most studies have their limitations and therefore there is still no final answer to the question, who should receive what type of adjuvant treatment. Merely in cases of uterine papillary serous carcinomas, which affect 1% to 10% of patients, there is consensus that patients should receive chemotherapy (with or without adjuvant radiotherapy) with a platinum-based regimen, combined with doxorubicin and cyclophosphamide. Newer regimens consider paclitaxel, with or without platinum [99].

### Adjuvant hormonal treatment

A multicenter, open, controlled, prospectively randomized trial on adjuvant endocrine treatment with medroxyprogesterone acetate (MPA) or tamoxifen in stage I and II endometrial carcinomas failed to detect differences in the disease-free and overall survival rates for a tamoxifen group compared with a control or a MPA group [100]. However, the total number of patients on trial (n = 388) seems too low in relation to the favorable prognosis of early stage disease and the low total response rate of tamoxifen which ranged around 10% in this situation [101]. In the aforementioned study tamoxifen demonstrated only modest activity which lead the authors to the conclusion that tamoxifen does not warrant further investigation as a single agent but perhaps a sequential use of tamoxifen and progestational agents [101].

### Adjuvant chemotherapy

The generally good prognosis of endometrial carcinomas does not justify a general recommendation of chemotherapy, especially in the early stages. Even so, patients at high risk (unfavorable histological type, deep myometrial invasion and lymph-vascular space involvement) seem to profit from adjuvant chemotherapy [102,103]. Studies on the subject unfortunately often lack a control arm so the effects of chemotherapy remain unclear [104]. Well designed studies in the group of high risk patients are warranted.

## Preservation of fertility

The development of endometrial cancer in young patients is usually related to unopposed estrogen stimulation. In patients with continuing a desire to have children, approaches have been made to preserve fertility. Primary hormonal therapy with progestins was suggested as an alternative treatment for surgery hereby offering the preservation of fertility. These must be interpreted with caution because of low case numbers and a publication bias. Treatment with megestrol acetate at 160 mg/day for 3 months or medroxyprogesterone acetate (MPA) at 200–800 mg/day for 2–14 months resulted in disease regression in 60 to 75%, however, the percentage of patients who actually delivered healthy children was much lower, ranging from 20 to 25% [105-107]. Furthermore, in several patients, persistent or recurrent disease was observed at the time of a later hysterectomy. In all these cases of unsatisfactory progestin therapy and delayed definitive surgical treatment may have adversely affected patient prognosis.

In essence, the problems regarding the optimal selection of patients for conservative progestin therapy are unsolved. Only cases with good prognostic factors are to be selected: Well differentiated tumors and tumors with known favorable prognoses (e. g. endometrioid type tumors) with no or minimal myometrial invasion and early stage disease. The greatest problem lies in the difficulty of appropriate staging, resulting in a potential underestimation of the problem. Maybe positron emission tomography, magnetic resonance imaging or a combination of both may be useful in this respect in due course.

Patients have to be carefully informed that this fertility preserving concept is still experimental. Moreover, these women must realize the low overall pregnancy rate which may partially be related to the origin of the disease (obesity, irregular menses, polycystic ovarian disease (PCOD) with chronic anovulation and infertility) [108]. Nowadays when treatment of infertility is frequently offered to elderly women, such conservative treatments should, however be investigated more thoroughly. Further questions concern follow-up of these patients. In the aforementioned studies, many patients were treated with maintenance therapy (oral contraceptives or cyclic progestins) to prevent recurrence, which was excluded by routine combination of sonography and D&C every 3–6 months.

## Palliative treatment – treatment of local recurrences

**Figure 6:**
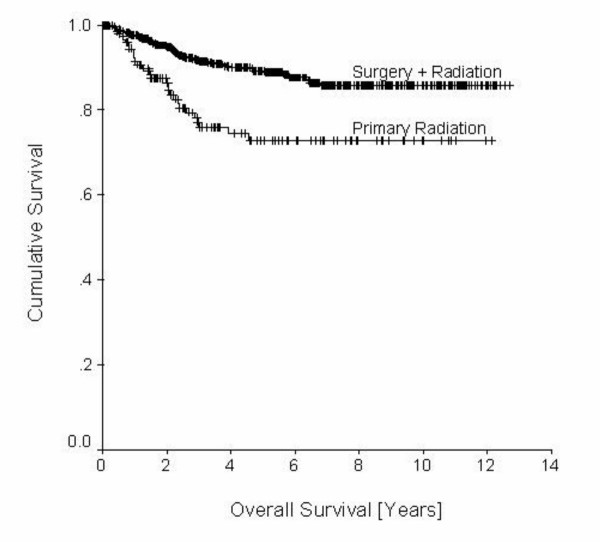
Comparison of overall survival between patients undergoing surgery and radiotherapy or primary radiotherapy. Note that patients in the group undergoing primary radiotherapy were generally older

Generally the prognosis for patients with recurrent disease is poor, therefore a thorough staging procedure should be performed. Analyses from other tumor entities, e.g. ovarian carcinomas, have demonstrated that a second surgical intervention may be useful in improving patients' overall survival [109,110]. Thus, even in absence of clinical studies on the subject, patients with single site recurrence should be evaluated for their suitability to surgery at relapse. In patients with isolated central recurrences, pelvic exenteration may be a potential option for cure [111]. The choice with respect to therapy of cancer recurrence strongly depends on prior treatment. In case of prior radiotherapy, a second intervention may often not be possible. If radiotherapy is possible, only nonbulky vaginal recurrences (< 0.5-cm thick) should be treated by intracavitary brachytherapy. Patients with bulky (> 0.5-cm thick) recurrences should receive interstitial techniques [94]. A general decision tree regarding the procedures in case of a recurrence is depicted in figure 6. Regarding the decision, whether or not to start with endocrine or cytotoxic treatment, the individual risk seems important. But equally important are aspects of patients' quality of life. A risk assessment scale, which was originally introduced by Possinger for breast cancer, seems helpful [108]. Similar to the adjuvant situation, treatment with tamoxifen, medroxyprogesterone acetate (MPA) alone or in combination may be used. However, there are only few, relatively old studies on the topic, all of which do not allow a final conclusion on the value of such therapies. MPA seems to be the best substance producing remission rates up to 80% in receptor positive tumors [113]. Remission rates of tamoxifen range around 25–30%. After failure of MPA, tamoxifen may be added to MPA producing remission rates in this combination of 50–60% [114]. Also aminogluthemide seems to be active in endometrial cancer [115]. Responses to all kinds of treatments unfortunately do not tend to last for long.

Some studies also suggest a potential benefit for luteinizing hormone-releasing hormone analogues (GnRH), although studies in endometrial carcinomas have not shown any convincing activity [116-118]. To sum, up on the background of the few and old studies with often low case numbers, newer large studies on endocrine strategies are warranted.

With respect to chemotherapy, several cyctostatic compounds have demonstrated efficiency. Among these are paclitaxel, carboplatinum, doxorubicin, cisplatin, etoposide, and 5 fluorouracil. Topotecan showed only limited activity [119-124]. All drugs may be used alone or in combination, perhaps even in combination with hormonal therapy [125,126]. Again, realizing that combination chemotherapy produces greater side effects, every option, endocrine treatment, single drug or combination chemotherapy should be considered.

## Follow-up

Generally recommendation for clinical follow-up of patients advocate patients to be monitored at three-monthly intervals during the first 3 years, at 6-monthly intervals up to the 5th year and at yearly intervals thereafter. Apart from provision of general information on all aspects of the disease and its treatment including unconventional cancer therapies, the documentation of patients' history, a clinical gynecological examination including a pap smear, a vaginal sonography and even the determination of tumor markers (SCC, CA125) have been recommended. Up to 95% of all recurrences may be detected early this way [127]. Again there are no prospective studies on the subject to enforce such recommendations. On the contrary, several retrospective studies indicate that an intensive follow-up does not result in a survival advantage for patients with recurrent disease but merely increases costs [7,128-131]. Also in this area the most appropriate management of endometrial carcinomas remains to be determined. In any case an annual examination of the breast, including mammography, is recommended, due to the fact of the frequent coincidence of malignancies of the breast [7].

## Estrogen replacement therapy in endometrial cancer patients

Hormone replacement therapy (HRT) with estrogen with or without progestins is frequently used to alleviate menopausal symptoms but also to reduce the risk of osteoporosis and cognitive dysfunctions. In endometrial carcinomas HRT may be believed to be critical due to fear of initiating growth of occult residual tumor cells, resulting in disease recurrence and shortened survival. As summarized by the American College of Obstetricians and Gynecologists (ACOG) there is not enough data to draw any final conclusion. Any decision on the subject should thus be individualized based on potential benefit and risk to the patient [132]. Some studies which have addressed this subject covered only a small number of patients in regard to the overall excellent prognosis of endometrial cancer patients. Of all these studies, however, none produced evidence that patients should not receive estrogen [133-136]. On the contrary, some studies reported an even prolonged survival for patients (with low-risk factors for recurrence, namely, low tumor grade (grades 1 and 2), less than 1/2 myometrial invasion, and no metastases to lymph nodes or other organs,) who received estrogen [134,136]. Furthermore, the introduction of selective estrogen receptor modulators (SERM) has also offered new treatment options which will also have to be studied in the future.

## Final remarks

Endometrial carcinomas represent a very frequent tumor entity in industrialized countries. It is hard to believe how little evidence-based data exists on even the important aspects of the disease. This may be partially due to the overall good prognosis even if surgery is reduced to hysterectomy and adnectomy. However, the high incidence in the developed world and consequently many women suffering relapses, necessitates the research of new approaches for cancer recurrence. Recent research suggests that therapy with trastuzumab (Herceptin^®^) could perhaps improve the outcome in HER-2/neu overexpressing tumors [137]. Further research will focus on molecules and pathways responsible for the initiation and growth of endometrial carcinomas, including tumor suppressor genes, DNA mismatch repair genes, oncogenes, molecules involved in adhesion and invasion and angiogenesis [138]. This research will hopefully allow the development of specific and selective inhibitors.

**Figure 7:**
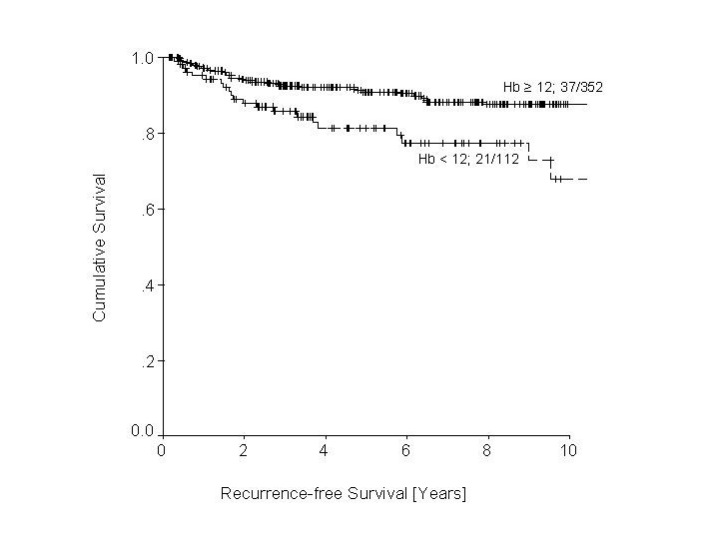
Comparison of recurrence-free survival between in patients undergoing adjuvant radiotherapy with respect to anemia. Log Rank = 9.1; df = 1; p < 0.003.

Some advances may also be possible with – conventional treatment, especially radiotherapy. Recent findings of a retrospective analysis suggest that tumor oxygenation may play an important role during adjuvant radiotherapy of endometrial carcinomas. Patients with normal hemoglobin levels during therapy (according to the definition of the EORTC = 12.0 g/dl) have a substantially better overall and recurrence free survival (Figure 7) [139]. Due to this strong impact on patients' health, these aspects require further studies.
